# Epigenetic Regulation of Dental Follicle Stem Cells in Odontogenic Regeneration

**DOI:** 10.1111/jcmm.70541

**Published:** 2025-04-28

**Authors:** Sibel Elif Gultekin, Leyla Arslan Bozdag, Margarete Odenthal, Hans‐Peter Dienes

**Affiliations:** ^1^ Department of Oral Pathology, Dental Faculty Gazi University Ankara Turkey; ^2^ Department of Biology, Faculty of Science Gazi University Ankara Turkey; ^3^ Institute for Pathology, Medical Faculty and University Hospital of Cologne University of Cologne Cologne Germany; ^4^ Medical University of Vienna Vienna Austria

**Keywords:** CD133, microRNA‐125, microRNA‐203, microRNA‐21, OCT4

## Abstract

MicroRNAs (miRNAs) are short non‐coding RNAs essential for biological functions that control the process of translation of mRNA into protein. The discovery of miRNAs in mesenchymal stem cells (MSCs), especially in odontogenic tissues and dental follicles, has not been fully characterised. This study focused on characterising dental follicle stem cells (DFSCs) in terms of their ability to proliferate and differentiate into osteoblasts using qRT‐PCR (miR‐203, miR‐125 and miR‐21) and immunohistochemistry (OCT4 and CD133). Dental follicles are essential for tooth eruption as they envelop the enamel organ and dental papilla and control the development and breakdown of the alveolar bone. Dental follicle progenitor cells (DFPCs) are stem cells located in dental follicles that differentiate into several cell types that are essential for tooth development and eruption. We observed that miR‐125 was upregulated in fibromyxoid and myxoid tissues during odonto/osteogenic differentiation of hDFPCs (fold change values, respectively, 1.75 ± 0.98 and 2.17 ± 1.03). miR‐203 and miR‐21 significantly downregulated odonto/osteogenic differentiation in myxoid, fibromyxoid and fibroid tissues (fold change values, respectively: miR‐203: 0.57 ± 0.25, 0.38 ± 0.11, 0.21 ± 0.18; miR‐21: 0.21 ± 0.14, 0.21 ± 0.13, 0.082 ± 0.14). Ultimately, utilising miRNA signatures in humans as a predictive tool will help us understand the molecular processes involved in DFSCs.

## 1 Introduction

Adult stem cells are excellent candidates for cell‐based therapy because they can produce differentiated cells and are self‐renewing [1]. Various tissues, including bone marrow, skin, liver, muscle and dental tissues, have reported their presence [2].

Human dental tissues are a potential source of tissue stem and progenitor cells, including pulp, periodontal ligament and human dental follicle cells (hDFCs). hDFCs originate from neural crest‐derived ectomesenchyme cells and are loose connective tissue sacs that surround the unerupted tooth, giving rise to three different cell types: cementoblasts, osteoblasts and fibroblasts, which form the cementum, alveolar bone and periodontal ligament. Their presence is required for eruption, whereby they appear to regulate the osteoclastogenesis and osteogenesis required for eruption [3].

Studies have demonstrated its potential as a source of multipotent stem cells capable of differentiating into non‐dental cell lineages like osteoblasts, adipocytes, hepatocytes and neurones [3, 4, 5]. hDFCs represent an alternative stem cell source because they are easily obtainable from extracted third molars, and approximately 70% of the human population has impacted third molars. Even though adult stem cells (also called multipotent stem cells or mesenchymal stromal cells) are used in medicine, the molecular processes that control their differentiation, growth and homeostasis are still not fully understood.

Since its identification in the early 1990s, Oct‐4 transcription factor expression has been proposed as a hallmark of pluripotency and a major gene in the regulation of embryonic differentiation. Oct‐4, sometimes referred to as Oct‐3 and Oct3/4, is a member of the POU family and is expressed in the germ line and pre‐gastrulation embryo [6, 7]. A specific threshold of Oct‐4 expression is essential for the self‐renewal and maintenance of totipotency in embryonic stem cells (ES) [8, 9]. Furthermore, varying levels of Oct‐4 expression trigger distinct developmental pathways, with a temporary elevation prompting commitment to primitive endoderm and mesoderm lineages, while repression results in trophectoderm differentiation [10]. At the conclusion of gastrulation, Oct‐4 is gradually downregulated and, by 8.5 days post coitum in mice, is only expressed in primordial germ cells. These data indicate a definitive function of Oct‐4 as a principal regulator of pluripotency [11].

Prominin‐1 was initially discovered in 1997 through two distinct research investigations examining murine neuroepithelial (NE) cells and human haematopoietic stem cells [12, 13]. Since its identification more than 20 years ago, numerous roles have been suggested for prominin‐1, including its function as a biomarker for stem cells and cancer stem cells (CSCs), its involvement in the organisation of plasma membrane protrusions, the maintenance of apical‐basal polarity in epithelial cells, the biogenesis of photoreceptive discs, mechanisms of multi‐drug resistance and its capacity for self‐renewal and tumour formation [14, 15, 16]. CD133 is not exclusively restricted to neuroendocrine progenitors; it is also present in both epithelial and non‐epithelial cell types. Throughout the developmental phases from the embryonic stage to maturity, this protein is expressed in many regions of the body [17].

**FIGURE 1:**
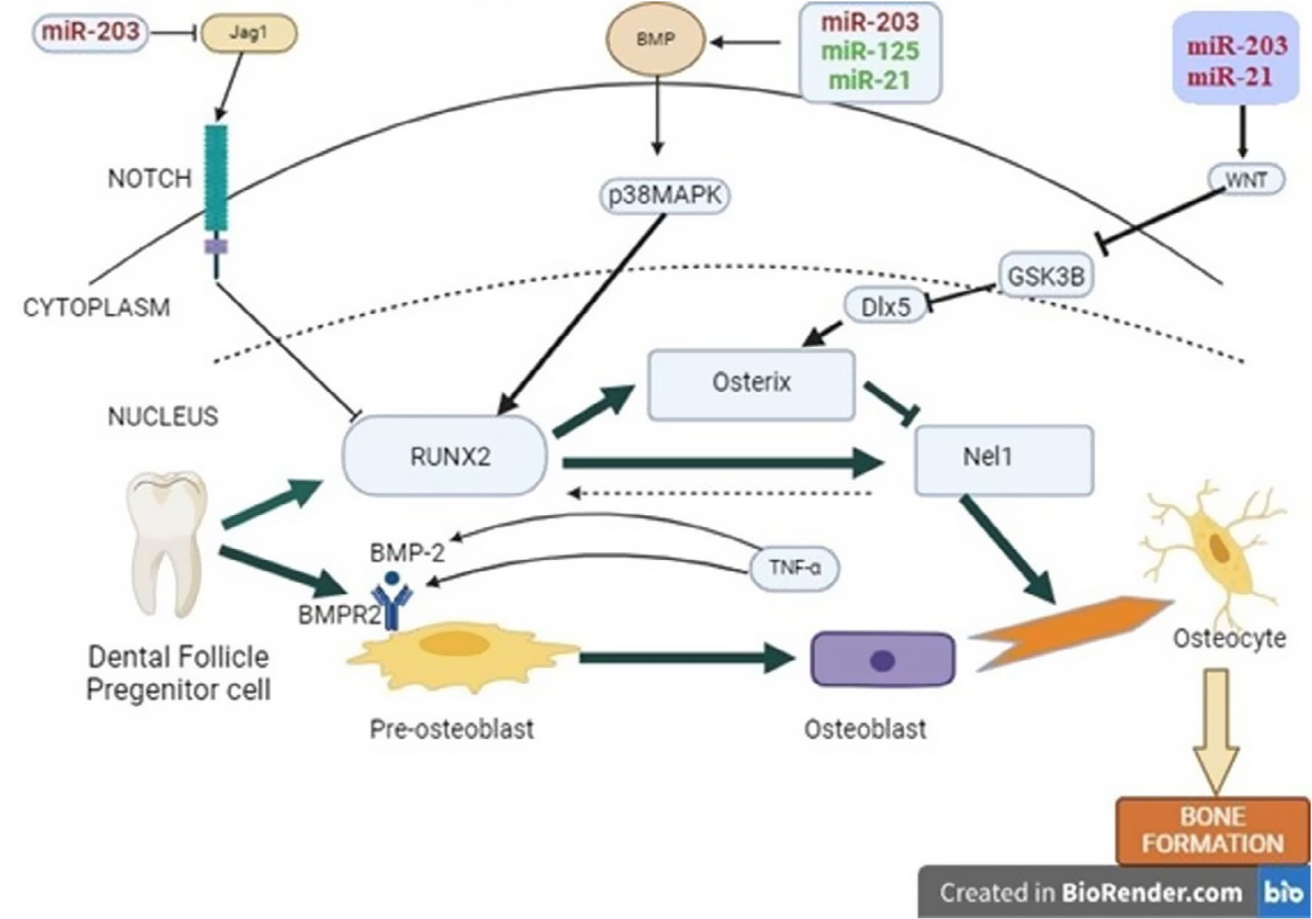
Role of miRNAs and signalling pathways in osteogenesis of MSCs. miRNAs inhibit (red) or promote (green) osteoblast development by targeting genes like Runx2 and osterix. Osteoblastic differentiation is regulated by BMP, Notch and Wnt pathways, involving key components such as Jagged 1 (Jag1), GSK‐β3, Dlx5 and MAPK.

The discovery of the post‐transcriptional control of microRNAs (miRNAs) has gained significant interest. Emerging evidence indicates that a class of single‐stranded noncoding RNAs, known as miRNAs, plays a critical role in this process [18]. Studies have extensively documented the impact of a large number of miRNAs on the development of mesenchymal stem cells (MSCs) and osteoblasts, highlighting the complex regulatory networks that govern cell fate determination [19, 20] (Figure 1). Regenerative medicine is a discipline of medical research that concentrates on stem cells to enhance human life quality by regenerating and repairing impaired organs [21]. Regenerative medicine and tissue engineering are methodologies that enable human cells, tissues or organs to regain their natural functionality. The process involves activating the body's repair mechanisms through stem cells to regenerate tissues or organs; when self‐healing is insufficient, laboratory‐grown tissues and organs are utilised to replace the damaged ones [22].

miR‐203 is a key regulator of epithelial differentiation, promoting differentiation by repressing stemness‐associated genes like p63. While its role in epithelial tissues is well established, recent studies suggest its involvement in mesenchymal differentiation processes as well, including osteogenesis. MiR‐203 impacts dental development by affecting the differentiation of dental cells [23]. Given that dental follicle stem cells (DFSCs) are of mesenchymal origin and play a crucial role in tooth development, investigating the specific targets and functions of miR‐203 in these cells is warranted [24]. In addition to its function in epithelial tissues, increasing evidence indicates that miR‐203 is also involved in mesenchymal development, particularly osteogenesis. Research indicates that miR‐203 can target Runx2, an essential transcription factor for osteoblast development. miR‐203 can influence the development of mesenchymal stem cells into osteoblasts via controlling Runx2 expression [24]. Additional study is required to thoroughly clarify the particular targets and downstream effects of miR‐203 in DFSCs, as well as their role in cementogenesis and other pertinent differentiation pathways.

Similarly, miR‐125 has emerged as a key regulator of stem cell differentiation and maintenance, influencing various pathways that determine cell fate. Its involvement in the differentiation of MSCs into osteoblasts makes it highly relevant for understanding the osteogenic potential of DFSCs [25]. Research indicates that miR‐125b expression progressively declines during BMP‐4‐induced osteoblastic differentiation of ST2 cells, which regulates breast cancer and neuronal cell proliferation and differentiation [25]. Furthermore, certain microRNAs, such as miR‐125 and miR‐21, have been demonstrated to be dysregulated in individuals diagnosed with periodontitis. Periodontitis is a condition marked by the deterioration of periodontal tissues coming from the dental follicle [26].

miR‐21 has been extensively studied for its role in modulating critical cellular processes such as proliferation, apoptosis and differentiation. Known for promoting osteoblast differentiation, miR‐21 is often associated with bone‐related regenerative processes, aligning with the study's focus on DFSCs and their ability to differentiate into osteoblasts [27]. By examining these specific miRNAs, the study aims to elucidate the molecular mechanisms underlying the differentiation potential of DFSCs, with implications for tooth development and bone regeneration. A stem cell‐specific miRNA or mRNA is expressed in undifferentiated stem cells, with its expression diminishing throughout differentiation into a specialised cell type. Embryonic stem cells exhibit elevated amounts of Oct4 mRNA, which diminish by over five‐fold upon differentiation. Furthermore, miR‐21 is expressed at elevated levels in embryonic stem cells relative to mature cells [28, 29]. Consequently, the expression patterns of miRNAs and mRNAs in immature cells differ from those in mature cells.

The role and expression pattern of these miRNAs in the differentiation of stem cells in dental tissues, including hDFCs, has not been well studied. A recent study has also identified the profile of miRNAs in mouse dental germs, suggesting that they are involved in the control of important developmental processes [30].

This study aimed to identify and characterise DFSCs by examining the expression of MSC surface markers. The expression of embryonic stem cell markers OCT4 and CD133 was evaluated in DFSCs using immunohistochemistry. RT‐qPCR was performed to quantify the expression of stem cell‐associated microRNAs (miR‐203, miR‐125 and miR‐21) in dental follicle tissues obtained by macrodissection and microdissection. Taken all together, the aim of this study is to characterise DFSCs by investigating their proliferative capacity and differentiation potential in terms of histological subgroups, which may indicate the maturation stage of the mesenchymal part of DFs. Furthermore, the study also evaluates the effect of miRNAs in odontogenesis.

## 2 Materials and Methods

### 2.1 Selection of the Dental Follicle Specimen

The study is conducted on a total of 30 formalin‐fixed, paraffin‐embedded (FFPE) tissue samples of dental follicles, which were obtained from the diagnostic tissue archive of the Department of Oral Pathology, Faculty of Dentistry, Gazi University, Ankara, Turkey. In the control group (*n* = 5), gingival fibroblasts without expected differentiation ability were used. The study samples were selected from archival material from 2008 to 2009. After the impacted teeth are surgically removed, the department routinely receives the material for possible pathology diagnosis. All individual participants included in the study provided informed consent. The Institutional Review Board of the Faculty of Dentistry at Gazi University, Ankara, approved the study protocol (no. 82‐09). The dental follicle specimens were reviewed on haematoxylin‐eosin‐stained slides under a light microscope by the oral pathologist (SEG). The cases that met the histological criteria that we had established were included in the study (Table 1). Data on cases, such as age and sex, were also recorded (Table S1).

**TABLE 1:** Histological selection criteria of samples and classification of dental follicle.

The histological selection criteria for the specimens were as follows	Histologic classification of the Dental Follicles according to the type of mesenchyme
(1) The lining epithelium does not show any proliferation or pathological alterations	(1) Myxoid Dental Follicle: Loose myxoid connective tissue with/without odontogenic epithelium
(2) The epithelium (if available) is non‐keratinised squamous or/and cuboid epithelium with one or a few layers	(2) Fibroid Dental Follicle: Dense collagenous connective tissue with/without odontogenic epithelium
(3) There should be no evidence of inflammatory infiltrate in the connective tissue	(3) Fibromyxoid Dental Follicle: Connective tissue with fibrous and loose myxoid areas with/without odontogenic epithelium.
(4) The connective tissue should have the characteristic appearance of mesenchymal tissue in the dental follicle, such as loose, fine collagenous and/or myxoid or fibrous connective tissue with or without odontogenic epithelial rests	

### 2.2 Tissue Preparation, Macrodissection and Microdissection

The study was carried out at the labs of both Institutions. Tissue preparation was performed on samples obtained from formalin‐fixed paraffin‐embedded (FFPE) blocks. This process includes the preparation of the slides for immunohistochemical staining, microdissection, macrodissection, RNA isolation, cDNA analysis and RT‐qPCR.

Tissues were fixed in 10% neutral buffered formalin (NBF) at room temperature for 24 h to ensure adequate preservation of morphology and nucleic acids while avoiding over‐fixation. After fixation, samples were dehydrated through a graded ethanol series, cleared in xylene and embedded in paraffin at 60°C. Sections of 5 μm thickness were cut using a microtome and placed on positively charged glass slides for microdissection and RNA isolation. FFPE blocks were stored at room temperature in a desiccated environment, while unstained sections were used within 2 weeks of cutting to minimise RNA degradation. RNase‐free tools and reagents were used throughout the process to maintain RNA integrity. In the study, we performed both macrodissection and microdissection procedures in order to provide specific tissues for the miRNA analysis.

Macrodissection is a technique used to isolate specific tissue regions or cellular populations from a larger sample using visual inspection or low‐magnification microscopy. It is typically performed on FFPE tissue sections and enables targeted genetic or molecular analysis of selected areas. In our study, FFPE sections were mounted on positively charged slides and stained with haematoxylin‐eosin (H&E) to visualise tissue structures. Macrodissection was utilised to collect myxoid and fibroid connective tissue samples, separately, by using a scalpel. Tissue material was macrodissected from unstained slides using H&E as a guide.

Microdissection is a sensitive technique used to isolate specific cells or microscopic regions from tissue sections, enabling highly targeted molecular analyses such as RNA or DNA extraction. This method, performed under a microscope, is particularly valuable for heterogeneous tissues. FFPE sections were typically stained with H&E to visualise cellular architecture. To achieve this objective, specimens exhibiting odontogenic epithelium were detected on H&E slides, and sections were extracted from the relevant blocks. Microdissection was conducted on 25 cases to extract the odontogenic epithelial rest islands and the follicular epithelium within the dental follicle tissue. All 30 specimens were utilised for macrodissection. We placed five‐micron (m)‐thick slices of FFPE tissue samples on glass slides covered with a 1.35‐m‐thick polyethylene membrane. The slides were also coated with 1% poly‐L‐lysine to help the tissue stick better. The sections were deparaffinised and stained with haematoxylin. We performed laser‐assisted microdissection using the Robot‐MicroBeam system (PALM) (Zeiss Axiovert 135, Germany). The odontogenic epithelium samples were taken from both epithelial rests in the connective tissue and lining epithelium of the dental follicle. The lining epithelial rest islands and lining epithelium were identified at 200× magnification under a microscope, and a group of cells was captured in each case (Figure S1). Subsequently, they were collected separately into Eppendorf caps containing 50 μL of Digestion Buffer (from the RNAlater kit). Microdissected tissues were stored in Digestion Buffer overnight at −80°C prior to RNA isolation.

### 2.3 Immunohistochemistry

Histological sections from paraffin‐embedded tissues were mounted on 4 μm adhesive slides for immunohistochemical staining. After deparaffinisation in xylene and rehydration through ethanol gradients, endogenous peroxidase activity was inhibited with Tris/H_2_O_2_ for 20 min. Antigen retrieval was performed before incubation. Primary antibodies anti‐Oct‐4 (1:50, rabbit polyclonal, Abcam) and anti‐CD133 (1:300, rabbit polyclonal, Abcam) were applied for 2 h. Slides were incubated with the EnVision Plus (Dako) detection system for 30 min, using diamine‐benzidine (DAB) as the substrate. Counterstaining was done with Mayer's haematoxylin. Positive controls included pluripotent human embryonic stem cells for anti‐Oct‐4 and colon cancer sections for anti‐CD133. Negative controls involved tissue treated with buffer only, without primary antibody. The semi‐quantitative classification was done under a light microscope at a magnification of 200 for both CD133 and OCT4.

Membranous and cytoplasmic staining for CD133 was considered positive. Staining of < 5% of cells was considered negative. Scoring was based on the extent of staining as indicated by the percentage of stained cells: 0, < 5% cell staining; 1, 5%–50%; 2, more than 50% cell staining [31].

Nuclear and cytoplasmic staining for OCT4 was considered positive. A staining index score of 4 or more is indicative of high Oct‐4 expression, while a score of 3 or less is indicative of low Oct‐4 expression [32].

### 2.4 RNA Isolation and cDNA Synthesis

RNA extraction was conducted on microdissected, macrodissected and/or whole FFPE tissue samples.

RNA was extracted from FFPE blocks via microdissection, followed by incubation with proteinase K at 65°C overnight. After centrifugation (13,000 RPM, 20 min), the samples were transferred, mixed with TRIzol and chloroform, and centrifuged again (15,000 RPM, 5 min). Glycogen and isopropanol were added, incubated at −20°C and centrifuged (15,000 RPM, 15 min). The pellets were dried at 37°C and resuspended in RNA‐free water. For macrodissected FFPE tissues, paraffin was removed using xylene and ethanol. An FFPE RNA isolation kit (Qiagen, Hilden, Germany) was employed for total RNA extraction after centrifugation and precipitation.

### 2.5 Reverse Transcription and Determination of miRNA Profile by Real‐Time PCR

RNA extracts were resuspended in 20 μL H_2_O, measured using an ND‐1000 NanoDrop spectrophotometer, and treated with DNAse and RNase inhibitor. Reverse transcription was carried out on 35 ng of RNA using the TaqMan microRNA reverse transcriptase kit. Equal amounts of cDNA were used for the real‐time amplification of the target genes (miR‐203, miR‐125 and miR‐21) according to the manufacturer's recommendations (Applied Biosystems), and miRNA levels were normalised to miRNA‐U6 as an internal control. U6 is a reliable internal control widely used in miRNA studies due to its stability, prevalence, size, structure and normalisation ability. The use of U6 enables accurate and reliable analysis of miRNA expression data. The 2–ΔΔ Ct method was employed to calculate miRNA expression levels [33].

### 2.6 Statistical Analysis

For all analyses, the conformity of the data to normal distribution was evaluated by the Shapiro–Wilk test. Non‐parametric tests were preferred because the data did not conform to normal distribution. The Kruskal‐Wallis test was used to compare microRNA levels and immunohistochemistry markers between three histological subgroups. We assessed the miRNA levels and immunohistochemistry findings in patients using the Kruskal‐Wallis test. Statistical analyses were performed on demographic data (age and gender) to evaluate group differences. The suitability of age, which is a continuous variable, to normal distribution was evaluated by the Shapiro–Wilk test. Since age data were not suitable for normal distribution, the Kruskal‐Wallis test was used to test the difference in age distribution between the groups. In cases where significant differences were found, pairwise comparisons were made with Dunn's test. Gender (male/female) was analysed between groups using the Chi‐Square Test. Fisher's Exact Test was preferred when cell frequencies were insufficient. Spearman Rho correlation analysis was conducted to assess the relationship between age and group. We performed other statistical analyses using GraphPad Prism 5 (GraphPad Software, La Jolla, CA, USA). Data are expressed as mean ± SD. *p < 0.05, **p < 0.01, ***p < 0,001.

## 3 Results

### 3.1 Evaluation of Demographic Data of Patients

A comparative analysis of the average age and gender distribution among myxoid, fibromyxoid and fibroid groups revealed that the myxoid group had the lowest mean age. Specifically, the mean age for the fibroid group was 19.75 ± 6.54 years, for the fibromyxoid group was 17.4 ± 4.08 years, and for the myxoid group was 15.25 ± 1.98 years. In terms of gender distribution, 71.4% of participants in the myxoid group were female, compared to 55.5% in the fibroid group and 57.1% in the fibromyxoid group. The findings of the study demonstrated no statistically significant differences or correlations in demographic factors across the histological subgroups (*p* > 0.05).

### 3.2 Histopathology

**FIGURE 2:**
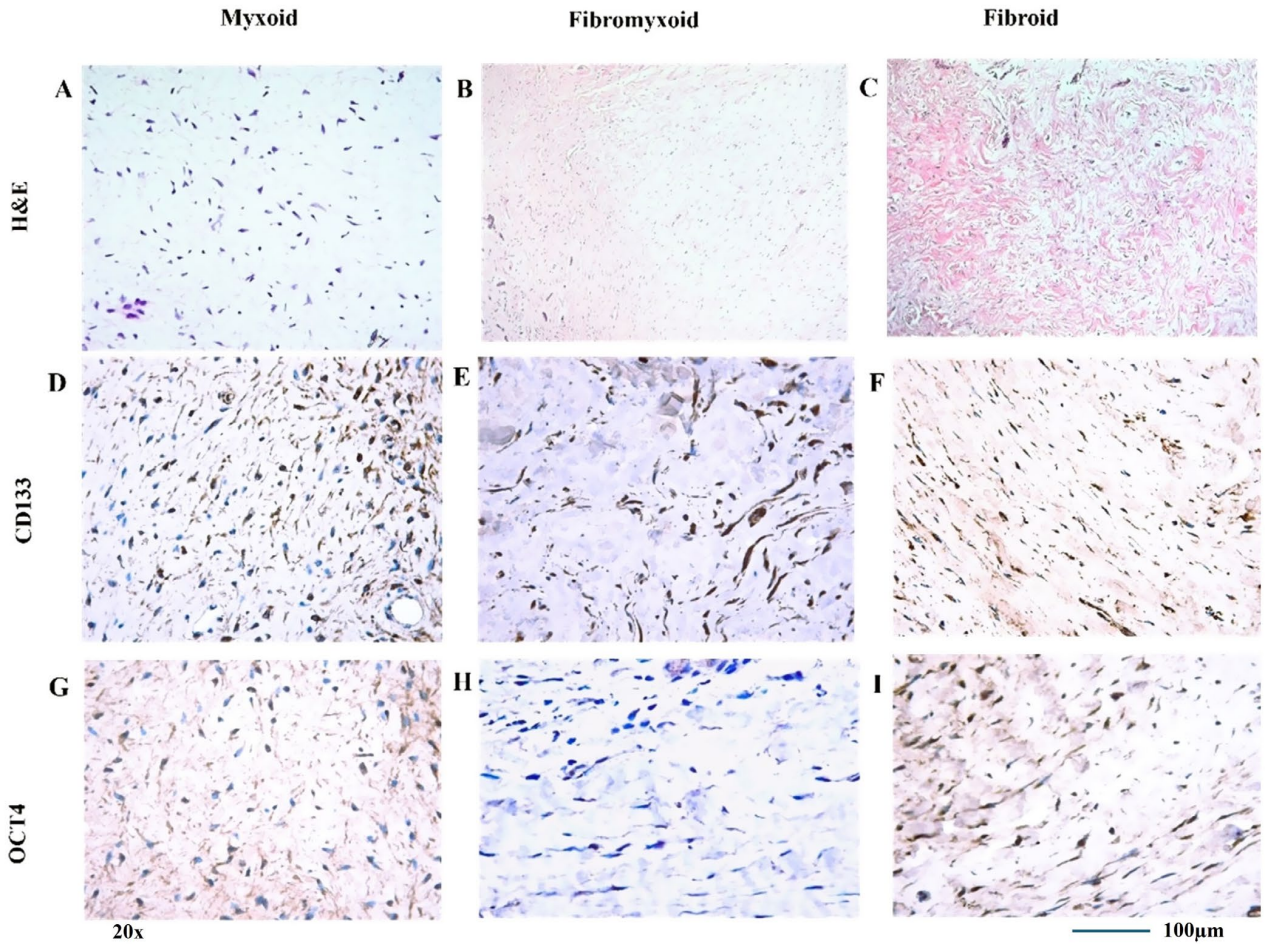
Staining of connective tissue types (myxoid, fibromyxoid and fibroid) with haematoxylin, eosin, CD133 and OCT4. Images exported from Leica QWin Plus v3.3.1; scale bar = 200 μm.

**FIGURE 3:**
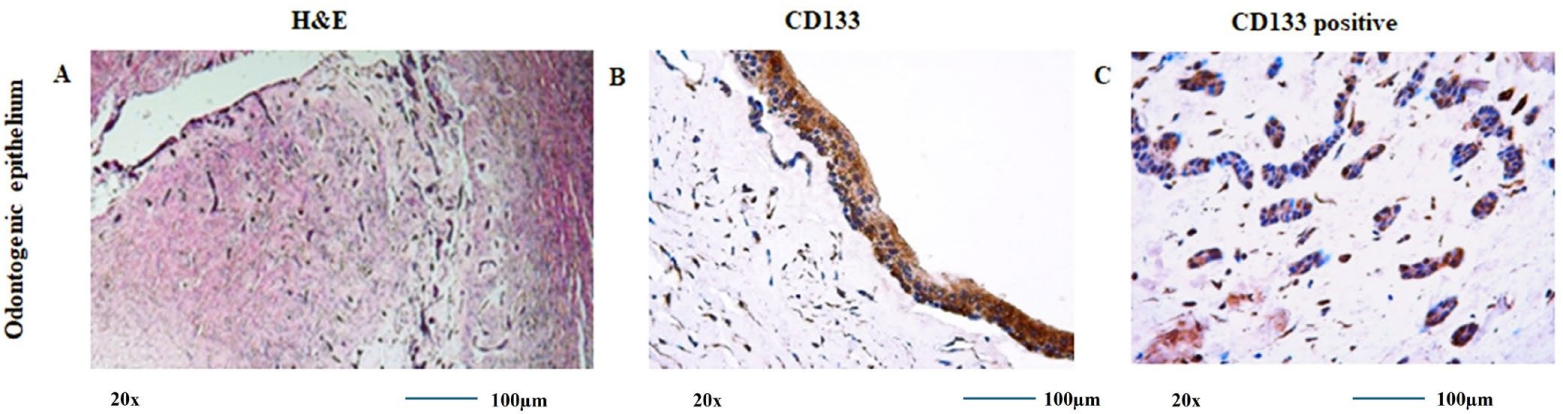
Staining of odontogenic epithelium using histochemical and immunohistochemical methods. (A) Haematoxylin‐eosin staining of odontogenic epithelium rest and lining. (B) CD133 positive immunohistochemical staining of odontogenic epithelium. (C) CD133 immunohistochemical staining in the nucleus and cytoplasm of the odontogenic epithelial lining. Images exported from Leica QWin Plus v3.3.1; scale bar = 200 μm.

The histopathological classification of the 30 DF samples was made according to the connective tissue stroma of dental follicles. Myxoid type: DF s with loosened cellular or hypocellular which is myxoid‐type, 7 samples (23.3%); fibromyxoid‐type, 14 samples (46.6%); and fibroid‐type, 9 samples (30%). Five of the 7 myxoid‐type DF samples had follicle epithelium and/or odontogenic epithelium rests (two cases with only epithelial lining one case with only odontogenic epithelial rests, two cases with both lining epithelium and odontogenic rests); 12 of 14 fibromyxoid DF samples showed epithelial lining; 10 had odontogenic epithelial rests; 7 of 9 fibroid DF samples had lining epithelium; and 8 of 9 DF samples contained odontogenic epithelial rests in the connective tissue. The lining follicle epithelium usually has one or a few layers of cuboid or non‐keratinised squamous cell epithelium. The mesenchymal (connective tissue) component of DF comprises spindle‐, plump‐, stellate‐ and tailed (myofibroblast‐like)‐shaped cells (Figures 2 and 3A).

### 3.3 Immunohistochemistry

This study investigated the presence of dental follicle progenitor cells within dental follicle (DF) samples by examining the expression of CD133 and OCT4 markers in both epithelial and mesenchymal components. CD133 expression was observed in cells of both tissue components, while OCT4 expression was infrequent in odontogenic epithelial and connective tissue cells (Figure 2).

CD133 staining was positive in various cell types: Lining follicle odontogenic epithelial cells, odontogenic epithelial rests, fibroblasts and fibroblast‐like cells in connective tissue. Fibromyxoid DFs showed the most frequent staining in the lining epithelium and connective tissue, whereas fibroid DFs were most frequently stained in the connective tissues (Figure 3B,C).

Odontogenic epithelium (both lining and rests) and fibroblasts showed positive staining for OCT4. The highest staining frequency for OCT4 was found in epithelial rests within fibroid and fibromyxoid DFs and in the connective tissue of myxoid DFs (Table S2).

Myofibroblast‐like cells in the connective tissue displayed stronger CD133 positivity compared to stellate and spindle‐shaped fibroblasts across all hDFC groups. The CD133 positive staining score for the lining follicle epithelium was higher in fibromyxoid DF tissue compared to fibroid DF tissue. Conversely, odontogenic epithelial rests in fibroid DF tissue exhibited a higher staining score than those in fibromyxoid and myxoid DF tissues. This study investigated the expression of CD133 and OCT4, markers associated with progenitor cells, in different types of DFs. The DFs were categorised into fibroid, fibromyxoid and myxoid. To assess differences in marker expression among these DF types, the non‐parametric Kruskal‐Wallis test was employed. The analysis revealed that there were no statistically significant differences in the expression levels of either CD133 or OCT4 among the fibroid, fibromyxoid and myxoid DF types (*p* > 0.05). This suggests that, within the scope of this study, the expression of these markers does not significantly vary depending on the DF type.

### 3.4 Determination of miRNA Expression Using RT –qPCR

#### 3.4.1 MiR‐203 Downregulation in Dental Follicle Cells

**FIGURE 4:**
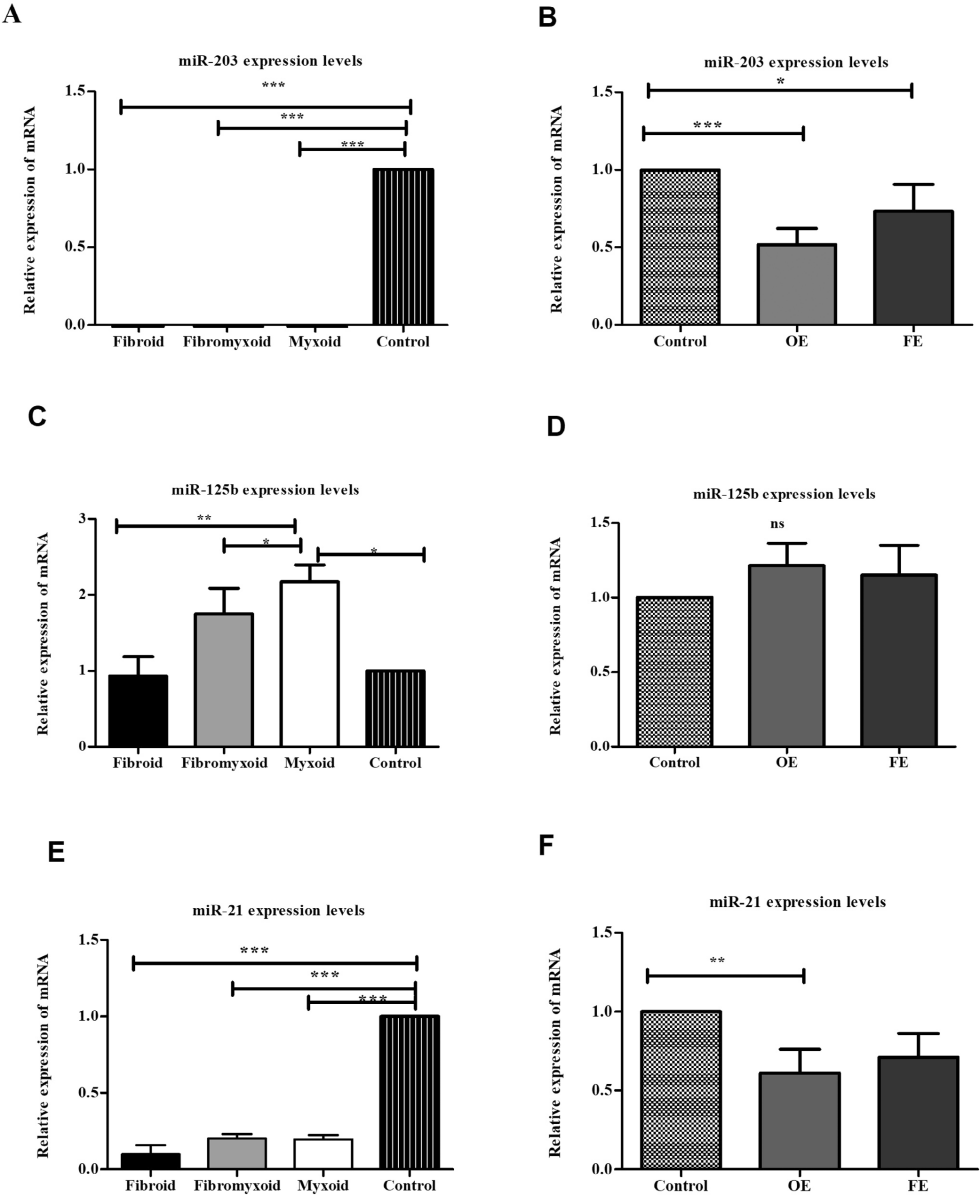
Histograms showing miRNA levels in different connective tissue types and the normal control group, determined by RT‐qPCR and normalised to miR‐U6. Histograms for macrodissected tissue (A, C, E) and microdissected tissue (B, D, F) are presented. Each RNA sample was tested in triplicate for accuracy (FE, fibroid epithelium; OE, odontogenic epithelium; *p < 0.05, **p < 0.01, ***p < 0.001).

**FIGURE 5:**
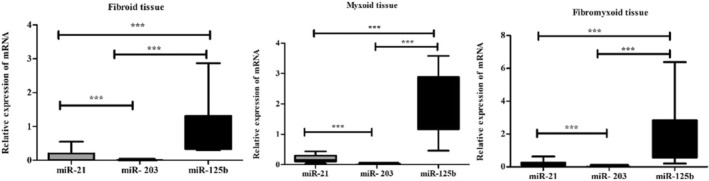
A comparison of groups obtained by macrodissection with microRNA.***p < 0.001.

The expression levels of microRNA‐203 were analysed in tissues obtained by macrodissection and microdissection. The data that did not display a normal distribution was subjected to a Kruskal‐Wallis test. A statistically significant difference was identified when fibroid, myxoid and fibromyxoid tissues obtained by macrodissection were compared with the control group (fold change values: 0.21 ± 0.18; 0.38 ± 0.11; and 0.21 ± 0.18, respectively; *p* < 0.001). Subsequent analysis compared microRNA expression levels between fibroid, fibromyxoid and myxoid histological subgroups. However, no statistical significance was identified between these subgroups (*p* > 0.05; Figure 4A; Table 2). The results demonstrated a decrease in the expression of microRNA‐203‐3p in fibroid, fibromyxoid and myxoid type dental follicles in comparison to the control group. Furthermore, the evaluation of miR‐203 expression in tissue samples collected by microdissection revealed a down‐regulation (*p* < 0.05) in both epithelial rest and lining epithelium (fold change: 0.51 ± 0.41 and 0.52 ± 0.39, respectively; Figure 5).

**TABLE 2:** Differential fold changes in miRNAs in hDFCs.

miRNA	Connective tissue types	Fold change/Standard deviation	Regulation	*p*
miR‐203	Myxoid (*n* = 7)	0.57 ± 0.25	Down	< 0.0001
	Fibroid (*n* = 9)	0.21 ± 0.18	Down	
	Fibro myxoid (*n* = 14)	0.38 ± 0.11	Down	
miR‐125	Myxoid (*n* = 7)^a^	2.17 ± 1.03	Up	0.0023
	Fibroid (*n* = 9)	1.0 ± 0.85	Up	
	Fibro myxoid (*n* = 14)	1.75 ± 0.98	Up	
miR‐21	Myxoid (*n* = 7)	0.21 ± 0.14	Down	< 0.0001
	Fibroid (*n* = 9)	0.082 ± 0.14	Down	
	Fibro myxoid (*n* = 14)	0.21 ± 0.13	Down	

^a^ MiR‐125b was significant in the myxoid group compared to other subtypes when comparing histological subgroups.

#### 3.4.2 MiR‐125b Upregulation in Dental Follicles

The expression levels of microRNA‐125b were analysed in tissues obtained by macrodissection and microdissection. The data that did not display a normal distribution was subjected to a Kruskal‐Wallis test. Our findings revealed that miR‐125 was significantly upregulated in myxoid, fibromyxoid and fibroid types of DFs (*p* < 0.05). Statistical significance (*p* < 0.05) was observed in all groups compared to the control group (fold change values: 2.17 ± 1.03; 1.75 ± 0.98; and 1.0 ± 0.85, respectively; Figure 4C). When histological subgroups were compared with each other, significant expression was found in the myxoid DF group (*p* < 0.0001). When miR‐125b expression was evaluated in tissue samples collected by microdissection, upregulation was found in both epithelial rests and lining epithelium (fold change: 1.55 ± 1.09 and 1.50 ± 1.11, respectively; *p* > 0.05; Figure 4D; Table S3). The data show statistically significant differences, and a positive correlation was found between the groups (*p* < 0.05, *r*: 0761). These findings suggest that miR‐125b may play a more important role than miR‐21 and miR‐203 in both odontogenic epithelium obtained by microdissection (Figure S2) and fibroid, fibromyxoid and myxoid tissues obtained by macrodissection (Figure 5).

#### 3.4.3 MiR‐21 Downregulation in Dental Follicles

We evaluated miR‐21 expression in fibroid, myxoid and fibromyxoid histological subtypes, as well as odontogenic epithelial remnants and lining odontogenic epithelium. miR‐21 expression was decreased in all three groups compared to the control group (fold change values: 0.21 ± 0.14; 0.21 ± 0.13; and 0.082 ± 0.14, respectively; *p* < 0.001; Figure 4E). No statistically significant difference was found between histological subgroups in terms of miR‐21 expression. When miR‐21 expression was evaluated in tissue samples collected by microdissection, upregulation was found in both epithelial rests and lining epithelium (fold change: 2.72 ± 4.61 and 2.83 ± 4.51, respectively; *p* < 0.05; Figure 4F). The Spearman correlation was not found between the immunohistochemical staining scores and RT‐qPCR (obtained by macrodissection tissues) expression values in any histological subtype of DFs.

## 4 Discussion

This study investigated the stem cell potential of DF tissue obtained from fully impacted teeth, focusing on the expression of specific miRNAs and their correlation with the histological classification of DFs. This classification, based on collagen fibre density and mucopolysaccharide content (fibroid, myxoid and fibromyxoid), represents different stages of DF maturation, impacting tissue differentiation, functional specialisation and the multipotent potential of dental follicle cells (DFCs) [34, 35, 36]. This study provides a novel histopathological classification of DFs, previously unavailable in the literature.

Human embryonic stem cells produce OCT4 transcription factors, which are crucial for establishing and preserving undifferentiated pluripotent stem cells [37, 38]. The importance of OCT4 expression in many cells is not well understood. Notably, only a small percentage of amniotic stem cells tested positive for OCT4 [39]. The absence of OCT4 expression in certain cells may be due to contamination with non‐stem cells. OCT4 is expressed in a mosaic pattern during normal development in a population of cells with equal differentiation potential [40]. Adult stem cells have limited differentiation capabilities compared to embryonic stem cells, which have the potential to develop tumours when transplanted into living organisms [41]. Although they are not derived from embryonic cells, approximately 10% of DFSCs express the fibroid‐type OCT4. Therefore, their capacity to distinguish is limited. Further in vivo experimental research is required to establish precise differentiation techniques and conduct a comprehensive evaluation of the behaviour of DFSCs before progressing to the clinical phase.

A recent study reported moderate expression of CD133 markers, previously identified in pulp and embryonic stem cells [42, 43]. CD133 plays a role in preserving stem‐like characteristics of cells by inhibiting differentiation and is thought to be associated with asymmetric division [44]. A recent study of stem cells in dental tissues found that dental follicle progenitor cells displayed Notch‐1, CD13, CD44, CD73, CD105 and STRO‐1 antigens but did not express CD34, CD45 or CD133 antigens, which are typically seen in bone marrow stem cells [45]. We detected myxoid, fibromyxoid and fibroid types in our hDF samples, but in minimal amounts. This indicates that hDF possess the ability to undergo differentiation, but to a limited extent.

The most striking finding was the significant upregulation of miR‐125 in the myxoid DF subtype (*p* < 0.0001). This is particularly interesting because the myxoid subtype, with its gelatinous extracellular matrix, resembles the primitive ectomesenchyme of the developing tooth [34]. We propose that this unique matrix composition may create a microenvironment that either promotes miR‐125 expression or is particularly sensitive to its regulatory effects. This heightened miR‐125 activity could be crucial for tightly controlling the balance between cellular proliferation and differentiation within this rapidly developing tissue. Given miR‐125's known roles in osteogenesis and cementogenesis, its upregulation in myxoid DF suggests a potential link between this subtype and the differentiation of DFCs into osteoblasts and cementoblasts, key cell types for bone and cementum formation. This finding supports the notion that myxoid DF tissue possesses a heightened stem cell potential. Furthermore, the observed upregulation of miR‐125 in both epithelial rests and lining epithelium suggests a broader role in epithelial‐mesenchymal interactions, which are essential for coordinated tooth development and eruption. This suggests that miR‐125 may be a critical modulator of signalling pathways involved in these complex processes.

Our study revealed that miR‐203‐3p expression is down‐regulated in DFCs. This decrease in miR‐203‐3p expression was associated with an increase in the expression of ALP and OCN [46], established markers of osteogenic differentiation, as evidenced by comparisons between control and the histologic subgroups. Given that miR‐203 is known to target Runx2 [46], a key transcription factor inhibiting osteoblast differentiation, this suggests that miR‐203‐3p may act as a negative regulator of these differentiation processes in DFCs. This is consistent with the established potential of hDFCs to serve as an important stem cell reservoir capable of differentiating into osteoblasts. The data further suggest that increased levels of miR‐203‐3p in all dental follicle subtypes may hinder their capacity for odonto/osteogenic differentiation. While these findings suggest a negative regulatory role for miR‐203‐3p, further studies, such as gain‐of‐function and loss‐of‐function experiments, are needed to confirm a direct causal relationship.

This contrasts with the role of miR‐125 and highlights the intricate interplay of different miRNAs in controlling DFC differentiation. Similarly, the reduced miR‐21 expression in hDFCs aligns with previous studies showing its inhibitory effect on osteogenic differentiation in human periodontal ligament stem cells (hPDLSCs) [27]. This consistency across different dental‐derived cell types reinforces the importance of miR‐21 as a key regulator of bone formation. The fact that miR‐21 inhibition promotes osteogenic differentiation and bone production in vivo further strengthens this conclusion and suggests potential therapeutic applications.

While miR‐21 and miR‐203 appear to play inhibitory roles in osteogenic differentiation within DFCs, miR‐125 emerges as a key positive regulator, particularly in the myxoid subtype. This distinct role for miR‐125 suggests it may be a critical molecular mediator in the osteogenic and cementogenic pathways of DFCs, influencing extracellular matrix components and cellular differentiation. This is particularly relevant given that DFCs are the source of cementoblasts, osteoblasts and fibroblasts essential for periodontal tissue formation [47]. Their demonstrated pluripotency, robust proliferative capacity and immunosuppressive properties [48, 49] make them promising candidates for regenerative therapies. Our findings regarding miRNA expression in distinct DF subtypes provide a more nuanced understanding of DFC differentiation potential and suggest that targeting specific miRNAs, particularly miR‐125, could be a promising strategy for enhancing tissue regeneration. Future studies should investigate the specific downstream targets of these miRNAs in DFCs and explore their therapeutic potential in preclinical models of periodontal regeneration.

DFCs possess superior immunomodulatory and anti‐apoptotic effects on the immune system compared to dental pulp stem cells (DPSCs) and stem cells from exfoliated deciduous teeth (SHED), and they also demonstrate enhanced osteogenic capabilities relative to SHED and DPSCs, as evidenced by the increased expression of osteogenic‐related markers such as Runx2 and DSPP in DFCs [50]. Furthermore, DFCs demonstrated the capacity to develop into cementoblasts in vivo [51]. Consequently, DFCs represent a promising alternative source for the regeneration of dental hard tissue. Yildirim et al. investigated the immunological effects of SHEDs, DPSCs and DFSCs on lymphocytes from healthy donors in vitro. They noted that DFSCs were readily obtainable and separated, demonstrating superior differentiation into several cell types compared to other dental MSCs. The DFCs, SHEDs and DPSCs inhibited lymphocyte proliferation, elevated Treg cell populations, reduced IL‐4 and IFN‐γ concentrations, and augmented IL‐10 levels. Furthermore, DFCs demonstrated an enhanced immunomodulatory effect on immune system cells. Following activation with IFN‐γ, DFCs demonstrated immunomodulatory capabilities, indicating their potential application in the treatment of autoimmune, inflammatory and allergy disorders [52].

## 5 Conclusion

In the context of DFCs, miR‐125 has emerged as a pivotal regulator of cellular differentiation, particularly in relation to the osteogenic potential of these cells. The upregulation of miR‐125 in specific histological subtypes of dental follicle tissues—myxoid, fibromyxoid and fibroid—provides important insight into its functional role in regulating cell behaviour in these distinct tissue environments.

Overall, the significant upregulation of miR‐125 in dental follicle subtypes, particularly in myxoid tissues, underscores its crucial role in the differentiation and regulatory pathways of these tissues. Its influence on cellular differentiation pathways, particularly those related to osteogenesis, offers valuable insights into its potential therapeutic applications in regenerative dentistry and tissue engineering, where modulation of miR‐125 could enhance the regenerative capacity of dental tissues.

## Author Contributions

**Sibel Elif Gultekin:** conceptualization (equal), funding acquisition (equal), investigation (equal), methodology (equal), project administration (equal), resources (equal), software (equal), supervision (equal), writing – review and editing (equal). **Leyla Arslan Bozdag:** conceptualization (equal), investigation (equal), validation (equal), visualization (equal), writing – original draft (equal). **Margarete Odenthal:** methodology (equal), writing – review and editing (equal). **Hans‐Peter Dienes:** validation (equal), writing – review and editing (equal).

## Conflicts of Interest

The authors declare no conflicts of interest.

## Supporting information


**Figure S1.** Laser‐assisted microdissection of lining odontogenic epithelium (A) and odontogenic epithelial rests (B: blue arrowhead) in dental follicle tissue.
**Figure S2**. A comparison of groups obtained by microdissection with microRNA (*p < 0.05, **p < 0.01).
**Table S1**. Statistical analysis of age and gender differences in groups.
**Table S2**. Mean of OCT4 and CD133 immunohistochemical scores (le: lining epithelium, er: epithelial rest, connective tissue).
**Table S3**. Differential fold changes in miRNAs via microdissection.

## Data Availability

The data supporting the findings of this study are available upon request from the corresponding author. The data were not publicly available because of privacy or ethical restrictions.
