# Supplementary material for: Epigenetic Regulation of Dental Follicle Stem Cells in Odontogenic Regeneration
Source: J Cell Mol Med. 2025 Apr 28;29(9):e70541. doi: 10.1111/jcmm.70541 (PMC12037698; doi:10.1111/jcmm.70541)
Supplement: Supplementary file 1 — Figure S1. Laser‐assisted microdissection of lining odontogenic epithelium (A) and odontogenic epithelial rests (B: blue arrowhead) in dental follicle tissue. Figure S2. A comparison of groups obtained by microdissection with microRNA (*p < 0.05, **p < 0.01). Table S1. Statistical analysis of age and gender differences in groups. Table S2. Mean of OCT4 and CD133 immunohistochemical scores (le: lining epithelium, er: epithelial rest, connective tissue). Table S3. Differential fold changes in miRNAs via microdissection. [file JCMM-29-e70541-s001.docx]

**EPIGENETIC REGULATION OF DENTAL FOLLICLE STEM CELLS IN ODONTOGENIC REGENERATION**

**Sibel Elif GULTEKIN^1^**[
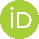
](https://orcid.org/0000-0002-0732-3617)**, Leyla ARSLAN BOZDAG ^1,2^**[
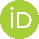
](https://orcid.org/0000-0002-4133-0319)**,** **Margarete ODENTHAL^3^**[
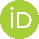
](https://orcid.org/0000-0002-2424-0960) **Hans-Peter DIENES^4^**

1. Gazi University Dental Faculty Department of Oral Pathology, Ankara, Turkey
2. Gazi University, Faculty of Science, Department of Biology, Ankara, Turkey
3. Institute for Pathology, Medical Faculty and University Hospital of Cologne, University of Cologne, Cologne, Germany.
4. Medical University of Vienna, Vienna -Austria

Corresponding author: Sibel Elif GULTEKIN ([sibelg@gazi.edu.tr](mailto:sibelg@gazi.edu.tr))


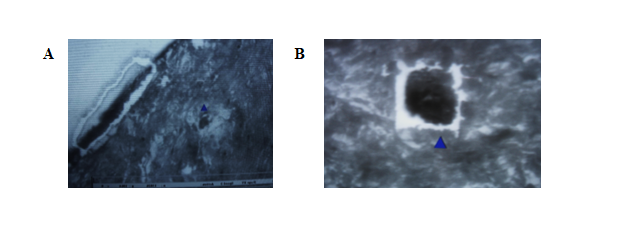


**Figure S1.** Laser-assisted microdissection of lining odontogenic epithelium (A) and odontogenic epithelial rests (B: blue arrowhead) in dental follicle tissue.


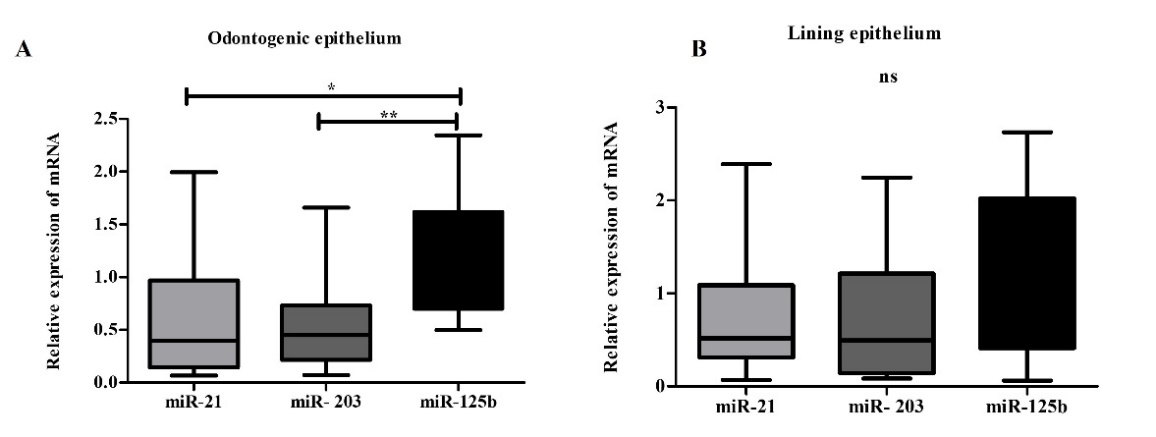


**Figure S2.** A comparison of groups obtained by microdissection with microRNA (*p < 0.05, **p < 0.01).

**Table S1.** Statistical analysis of age and gender differences in groups

| **Demographic values** | **Control (n=5)** | | **Myxoid (n=7)** | | **Fibroid (n=9)** | | **Fibromyxoid**  **(n=14)** | | ***p* value** | ***r correlation*** |
| --- | --- | --- | --- | --- | --- | --- | --- | --- | --- | --- |
|  | **n** | **%** | **n** | **%** | **n** | **%** | **n** | **%** | 0,51 | -0,866 |
| **Female** | 4 | 80 | 5 | 71,4 | 5 | 55,5 | 8 | 57,1 |  |  |
| **Male** | 1 | 20 | 2 | 28,6 | 4 | 44,5 | 6 | 42,9 |  |  |
| **Age** | **Mean** | **S.D.** | **Mean** | **S.D.** | **Mean** | **S.D.** | **Mean** | **S.D.** | ***p* value** | 0,500 |
|  | 40,8 | 10,5 | 15.25 | 1,98 | 19,75 | 6,54 | 17,41 | 4,08 | 0,88 |  |

| **Histologic sybtypes** | **Oct4le** | **Oct4er** | **Oct4ct** | **Cd133le** | **Cd133er** | **Cd133ct** | ***P* value** |
| --- | --- | --- | --- | --- | --- | --- | --- |
| Myxoid (n=7) | 0 | 0 | 0,3 | 0,6 | 0,6 | 1,3 | 0,06 |
| Fibromyxoid (n=14) | 0,2 | 0,4 | 0 | 1,5 | 1,3 | 1,6 |  |
| Fibroid (n=9) | 0,8 | 1,1 | 0,8 | 1,3 | 1,4 | 1,5 |  |

**Table S2**. Mean of OCT4 and CD133 immunohistochemical scores (le: lining epithelium, er: epithelial rest, connective tissue)

**Table S3.** Differential fold changes in miRNAs via microdissection

| **miRNA** | **Odontogenic epithelial types** | **Fold change/Standard deviation** | **Regulation** | ***p* value** |
| --- | --- | --- | --- | --- |
| miR-203 | Lining epithelium | 0,52±0,39 | Down | **0,0004** |
|  | Odontogenic epithelial rests | 0,51±0,41 | Down |  |
| miR-125 | Lining epithelium | 1,50±1,11 | Up | 0,86 |
|  | Odontogenic epithelial rests | 1,55±1,09 | Up |  |
| miR-21 | Lining epithelium | 2,72±4,61 | Down | **0,0065** |
|  | Odontogenic epithelial rests | 2,83±4,51 | Down |  |
